# A protease-based biosensor for the detection of schistosome cercariae

**DOI:** 10.1038/srep24725

**Published:** 2016-04-19

**Authors:** A. J. Webb, R. Kelwick, M. J. Doenhoff, N. Kylilis, J. T. MacDonald, K. Y. Wen, C. McKeown, G. Baldwin, T. Ellis, K. Jensen, P. S. Freemont

**Affiliations:** 1 Centre for Synthetic Biology and Innovation, Imperial College London, London, UK; 2 Section of Structural Biology, Department of Medicine, Imperial College London, London, UK; 3 School of Life Sciences, University of Nottingham, Nottingham, UK; 4 Department of Life Sciences, Imperial College London, London, UK; 5 Department of Bioengineering, Imperial College London, London, UK

## Abstract

Parasitic diseases affect millions of people worldwide, causing debilitating illnesses and death. Rapid and cost-effective approaches to detect parasites are needed, especially in resource-limited settings. A common signature of parasitic diseases is the release of specific proteases by the parasites at multiple stages during their life cycles. To this end, we engineered several modular *Escherichia coli* and *Bacillus subtilis* whole-cell-based biosensors which incorporate an interchangeable protease recognition motif into their designs. Herein, we describe how several of our engineered biosensors have been applied to detect the presence and activity of elastase, an enzyme released by the cercarial larvae stage of *Schistosoma mansoni.* Collectively, *S. mansoni* and several other schistosomes are responsible for the infection of an estimated 200 million people worldwide. Since our biosensors are maintained in lyophilised cells, they could be applied for the detection of *S. mansoni* and other parasites in settings without reliable cold chain access.

Millions of people worldwide are affected by parasitic diseases which confer a high healthcare cost and burden, especially in resource-limited settings. To break this disease cycle and enable the local eradication of these parasites, rapid and cost-effective methods of detection are desirable. A common biological signature of parasitic diseases is the production and release of specific proteases by the parasites at multiple stages in their life cycles^1^. Parasites and their eggs typically secrete proteases in order to invade their hosts, evade host defences and digest the local environment to provide nutrients^1^. The emerging field of synthetic biology is at the forefront in providing cutting-edge technologies and novel approaches^2^ for the development of biosensors. To this end, we explored synthetic biology approaches that could be applied towards the detection of parasites, and in this study we have engineered a modular whole-cell-based biosensor platform that can rapidly detect different parasites via their protease signatures. As a proof of concept, we have designed, built and tested several whole-cell-based biosensors that specifically target the parasite *Schistosoma mansoni*.

*S. mansoni*, like other members of the *schistosoma* genus of fluke worms, is a causative agent of the debilitating disease schistosomiasis (or bilharzia). Estimates suggest that over 200 million people worldwide are infected^3^,^4^. Indeed, the annual mortality rate for this disease is thought to be upwards of 280,000 people in sub-Saharan Africa alone^4^. The high infective rate of these parasites is due in part to their complex life cycle. After mating inside the human host, the female adult worm produces hundreds to thousands of eggs (depending on the species) per day^4^. The eggs are excreted from the host in the urine or faeces, and when contact is made with water the eggs hatch releasing miracidia^5^. These larvae then hunt for the intermediate host, freshwater snails^4^. After penetration of the snail, the parasites multiply and develop into cercariae^4^. The cercariae are released by the snails and can survive for 8–72 hours in the aquatic environment whilst they search for a suitable host^4^. In the case of schistosomes, such as *S. mansoni*, which infect humans, the cercariae are phototaxic and thus move towards the surface of shallow waters where they can maximise their chance of contact with humans^5^. The cercariae also follow a thermal gradient to find their potential hosts, and when they make contact with human skin, the presence of chemical signals, including medium-chain fatty acids such as linoleic acid, act as a stimulator for skin invasion^5^. The first step in the invasion process is the release of gland contents from the acetabular gland complex of the posterior of the cercarial head^5^. One of the factors secreted from the glands is serine protease activity, including a cercarial elastase, which facilitates invasion by degrading the dermal elastin^6^. Cercarial elastase activity has been attributed to a composite of the activities of a number of isoforms of one enzyme^7^. Furthermore, positional scanning – synthetic combinatorial library screening has identified a specific recognition motif for this elastase activity from *S. mansoni* cercariae^7^. Building upon these insights we hypothesised that the detection of *S. mansoni* cercarial elastase-specific protease activity would provide a novel approach for the rapid detection of *S. mansoni*. To this end, we engineered several modular, whole-cell-based biosensors which incorporate an interchangeable protease recognition motif into their designs. Proteolytic cleavage at the recognition motif results in the removal of a labelling region and thus provides detection via a ‘loss of colour’. To validate our approach, we initially used a synthetic biology approach to engineer several *Escherichia coli* and *Bacillus subtilis* whole-cell biosensors that specifically recognise TEV protease activity. We chose these two organisms as hosts since *E. coli* can be used to rapidly develop biosensors in the laboratory, whilst *B. subtilis* is classified as a generally recognized as safe (GRAS) organism by the American Food and Drug Administration for certain applications^8^, and thus gives us the possibility of using the *B. subtilis* based whole-cell biosensors *in situ*. With the rationale of the TEV-biosensor designs validated using both of these hosts, we wanted to examine whether our elastase-specific biosensor designs could detect elastase activity in secretions derived from cercariae that had been shed from infected snails. Here we report, that both our *E. coli* and *B. subtilis* elastase-specific biosensors successfully detected elastase activity from these biological samples and furthermore, we demonstrate that the plasmids encoding our biosensors are maintained in lyophilised *B. subtilis* cells, and therefore our biosensors were suitable for the detection of *S. mansoni,* as well as other parasites in settings that do not have reliable cold chain access. To our knowledge, these are the first whole-cell-based biosensors that have been directly designed for the detection of *S. mansoni* and this offers the possibility of developing further whole-cell-based biosensors for other parasites.

## Results

### Design specification and rationale of the protease biosensors

**Figure 1:**
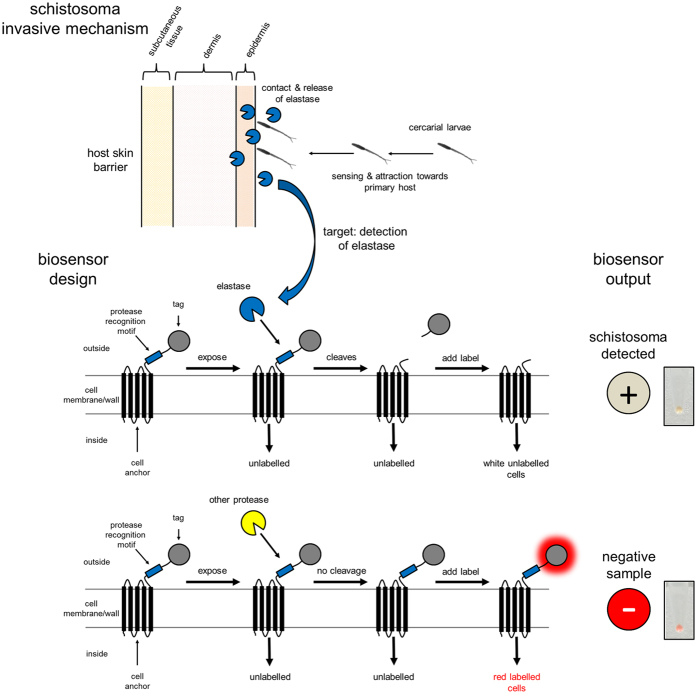
Detection of *Schistosoma mansoni* via cercarial elastase activity. *S. mansoni* cercariae secrete elastase which enables the parasite to penetrate the skin barrier and invade its hosts. In the example shown in this figure our Schistosoma biosensor has been designed to detect cercarial elastase activity. Our engineered whole-cell biosensors incorporate an interchangeable protease recognition motif into their designs. Proteolytic cleavage at the recognition motif via the activity of a specific enzyme results in the removal of a labelling region and thus provides detection via a ‘loss of colour’.

**Figure 2:**
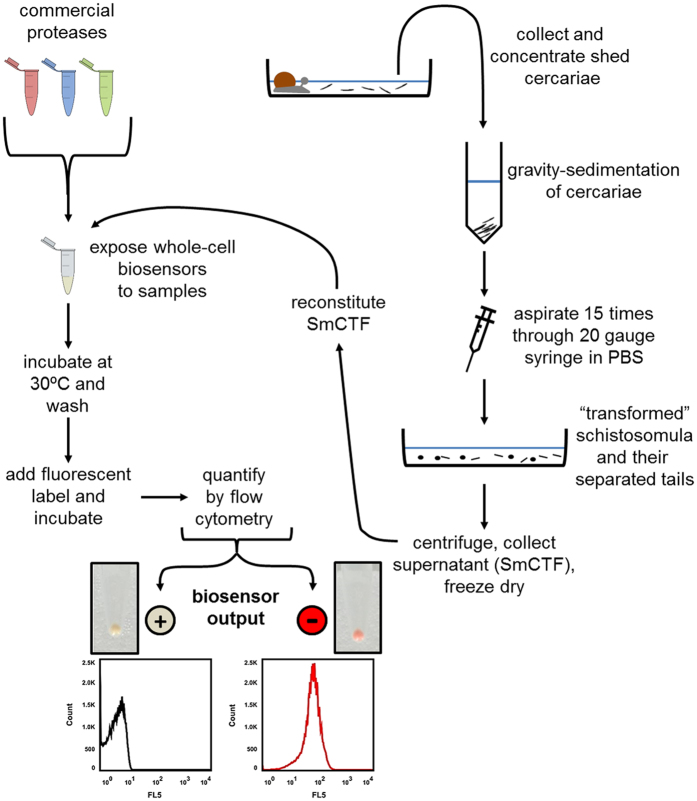
Schistosoma biosensor work-flow. *S. mansoni* cercariae are shed from infected snails and mechanically-transformed to produce *S. mansoni* cercarial transformation fluid (SmCTF) samples, which were lyophilised. Whole-cell biosensors are treated with either reconstituted SmCTF biological samples or a control protease from a commercially sourced panel. Whole-cell biosensors are washed and labelled. Labelled cells are analysed via flow cytometry. Proteolytic cleavage of the biosensor via the activity of SmCTF-derived cercarial elastase prevents cell labelling, thus resulting in a ‘loss of colour’ biosensor output (+detection of *S. mansoni*). Labelled cells indicate that the biosensor was not proteolytically cleaved thus resulting in a ‘gain of colour’ biosensor output (−detection of *S. mansoni*).

For a whole-cell-based biosensor to be functional and detect its specific target in the external environment, the biosensor component itself needs to be localised on the cell surface such that it is exposed to the target. Our biosensors have been designed to target the elastase activity released by *S. mansoni* cercariae to facilitate their invasion into their primary host: humans (Fig. 1). The biosensors have two general modular components: 1) an anchor module to localise and bind the sensor on the cell and 2) a detection module that has specificity for the target of the sensor (Fig. 1). The detection module comprises flexible linkers, the specific recognition motif of the protease that the sensor targets and an epitope tag for detection. When the biosensor is expressed in the host cell and localised to the outer membrane (*E. coli*) or cell wall (*B. subtilis*) in the correct orientation, the detection module is exposed to the external environment. Exposure of the biosensor to the protease specific for the recognition motif results in the removal of the epitope tag (Fig. 1). The fluorescent label that recognises and interacts with the epitope tag is therefore unable to bind to the biosensor and the cells appear colourless, thereby giving us a positive detection output (Figs 1 and 2). If the biosensor is exposed to a protease that does not recognise the detection module the motif is not cleaved, leaving the epitope tag still attached (Fig. 1). The fluorescent label is thus able to bind to the biosensor and the cells appear red in colour resulting in a negative detection output (Figs 1 and 2).

We have two biosensor systems, one housed in *E. coli* and the other housed in *B. subtilis*. For both hosts three biosensors were designed: one with specificity for the Tobacco Etch Virus (TEV) protease (TEV; motif -ENLYFQG-)^9^,^10^,^11^,^12^, one with specificity for cercarial elastase (ELA; motif -SWPL-)^7^, and one with a control motif (CON; motif -GSSQSG-). The TEV sensors act as positive controls in our study and validate our approach as the action and specificity of this protease has been well studied^9^,^10^,^11^,^12^. The CON sensor acts as a negative control as the recognition motif is a neutral sequence that should not be recognised by proteases. The amino-acid sequences of the *E. coli* and *B. subtilis* biosensor designs are shown in Supplementary Table 1.

**Figure 3:**
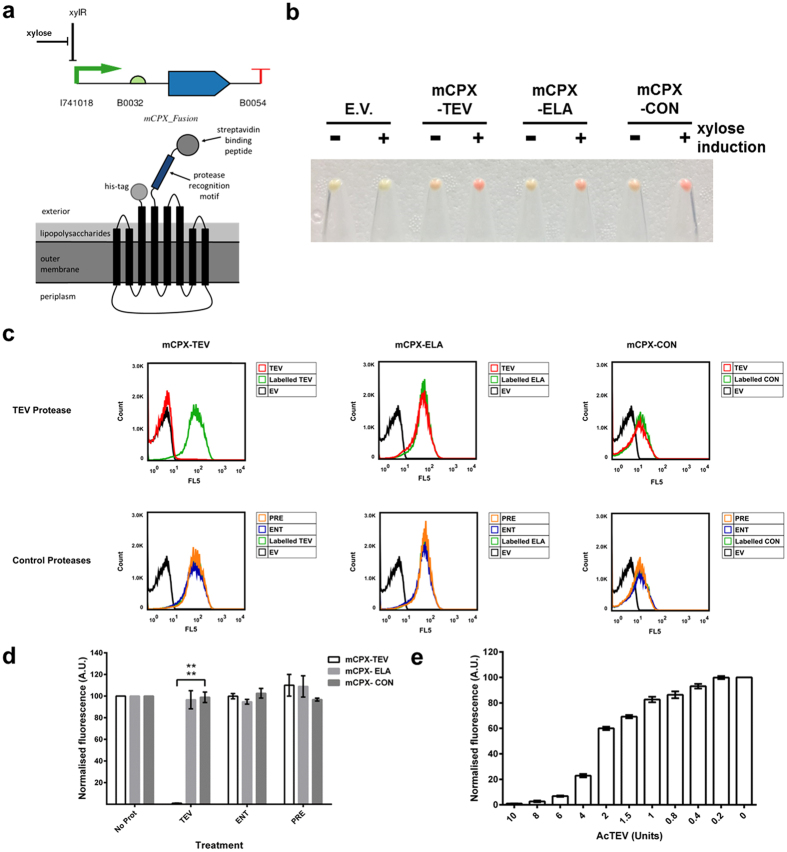
Validation of *E. coli* whole-cell biosensors. (**a**) Biosensor circuit design and localisation in the outer membrane of the cell. (**b**) Induction of biosensor expression. Representative cell pellets (OD_600_ 4.0) of either induced 100 mM xylose (+) or uninduced (−) biosensor-expressing cells were labelled with 1.25 μg streptavidin-R-phycoerythrin (SAPE)-conjugated antibody. Cell labelling (Red) indicates appropriate expression and localisation of the whole-cell biosensor. (**c**) Flow cytometry analysis of whole-cell biosensors. *E. coli* expressing either TEV (mCPX-TEV), elastase (mCPX-ELA) or control (mCPX-CON) biosensors were treated with the indicated proteases: AcTEV protease (TEV) or control proteases - PreScission protease (PRE) or Enterokinase (ENT). Treated cells were labelled with SAPE-conjugated antibody and analysed by flow cytometry. Labelled, non-protease treated cells and *E. coli* transformed with an empty vector plasmid (EV) served as experimental controls. (**d**) Summary of flow cytometry data. *E. coli* expressing either mCPX-TEV, mCPX-ELA or mCPX-CON biosensors were treated with the indicated proteases. The fluorescence (Geometric mean FL5) of protease treated cells were normalised against labelled, non-protease treated cells (No Prot). These data were analysed using FlowJo (vX 10.0.7r2) software and are representative of three independent biological repeats. (**e**) Sensitivity of mCPX-TEV biosensor. *E. coli* expressing mCPX-TEV biosensor were treated with 0–10 Units of AcTEV and analysed via flow cytometry. These data are normalised against untreated (0 U) labelled cells and represent the mean geometric mean ± the standard deviation of three independent experiments. Student t-test ****P < 0.0001.

The design of the *E. coli* housed whole-cell biosensor is based on that previously used in a study to identify protease recognition specificities using cellular libraries of peptide substrates (CLiPS)^13^,^14^. The CLiPS system uses the circularly permuted version of the *E. coli* outer membrane protein OmpX, CPX, as the anchor module^13^,^14^. CPX has been engineered such that both its N- and C-termini are exposed to the exterior environment^14^. A poly-histidine tag is located on the C-terminal whilst the protease detection module with the streptavidin-binding peptide (WCHPMWEVMCLR)^13^ epitope tag is present on the N-terminus (Fig. 3a). The three *E. coli* housed biosensors employed in this study (mCPX-TEV, mCPX-ELA and mCPX-CON) were created by replacing the recognition motif present in the CLiPS system with the relevant peptide sequence as described in the materials and methods section. The biosensor fusion genes were placed under the control of the xylose inducible promoter *xylF* (BBa_I741018) and the moderately strong RBS B0032 (BBa_B0032)^15^ as shown in the circuit visualised using Pigeon^16^ (Fig. 3a). This was to avoid any potential deleterious effects caused by overexpression of this outer-membrane protein by the cells. Furthermore, it was decided that the *E. coli* cloning strain NEB-10beta would be the host of choice for the biosensors, as this is not classically an expression strain and so, theoretically, would not overexpress the biosensors and would therefore minimise the risk of any detrimental burden-based responses.

**Figure 4:**
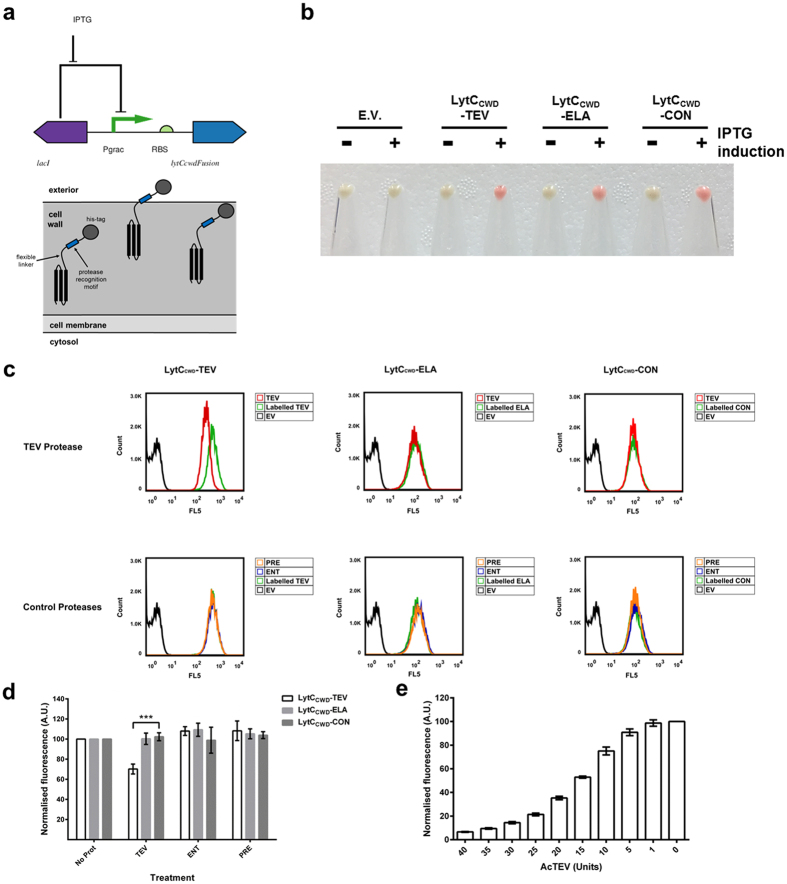
Validation of *B. subtilis* whole-cell biosensors. (**a**) Biosensor circuit design and localisation in the cell wall. (**b**) Induction of biosensor expression. Representative cell pellets (OD_600_ 4.0) of either induced 1 mM IPTG (+) or uninduced (−) biosensor-expressing cells were labelled with 2.5 μg His-phycoerythrin (His-PE)-conjugated antibody. Cell labelling (Red) indicates appropriate expression and localisation of the whole-cell biosensor. (**c**) Flow cytometry analysis of whole-cell biosensors. *B. subtilis* expressing either TEV (LytC_CWD_-TEV), elastase (LytC_CWD_-ELA) or control (LytC_CWD_-CON) biosensors were treated with the indicated proteases: AcTEV protease (TEV) or control proteases - PreScission protease (PRE) or Enterokinase (ENT). Treated cells were labelled with His-PE-conjugated antibody and analysed by flow cytometry. Labelled, non-protease treated cells and *B. subtilis* transformed with an empty vector plasmid (EV) served as experimental controls. (**d**) Summary of flow cytometry data. *B. subtilis* expressing either LytC_CWD_-TEV, LytC_CWD_-ELA or LytC_CWD_-CON biosensors were treated with the indicated proteases. The fluorescence (Geometric mean FL5) of protease treated cells were normalised against labelled, non-protease treated cells (No Prot). These data were analysed using FlowJo (vX 10.0.7r2) software and are representative of three independent biological repeats. (**e**) Sensitivity of LytC_CWD_-TEV biosensor. *B. subtilis* expressing LytC_CWD_-TEV biosensor were treated with 0-40 Units of AcTEV and analysed via flow cytometry. These data are normalised against untreated (0 U) labelled cells and represent the mean geometric mean ± the standard deviation of three independent experiments. Student t-test ***P < 0.001.

We also chose *B. subtilis* as a host for our biosensors as it is a well-studied and widely utilised organism that has successfully been used to overexpress many useful compounds and antibodies^17^,^18^,^19^, is generally recognised as safe by the FDA^8^ and has been used as a host for the surface localisation of enzymes including recombinant lipases such as the *B. subtilis* lipase B (LipB)^20^ and the *Aspergillus oryzae* lipolytic enzyme CutL^21^. Indeed, the binding of LipB to the surface of this bacterium was accomplished by fusing it to the N-terminal cell wall-binding domain of the native *B. subtilis* autolysin CwlB (LytC)^20^,^22^. To this end, we decided to incorporate the cell wall-binding domain of *B. subtilis* LytC into our biosensor design, and use it as the cell anchor module (Fig. 4a). This is not the only difference between the *B. subtilis* biosensor design and that of the *E. coli* biosensors. Unlike the *E. coli* based biosensor, the *B. subtilis* based biosensors only have their C-termini exposed to the external environment. Furthermore, the detection module for the *B. subtilis* based biosensors differs slightly in that the epitope employed is a poly-histidine tag. However, the three recognition motifs were identical and this resulted in the construction of the three biosensors LytC_CWD_-TEV, LytC_CWD_-ELA and LytC_CWD_-CON.

We decided to clone the fusion genes encoding the *B. subtilis* housed biosensors into a non-integrative plasmid to increase the expression levels of the biosensors. However, non-integrative plasmid maintenance and stability can be problematic in *B. subtilis*^19^. This issue has been resolved by using plasmids that use the θ-mode of replication^23^,^24^. Therefore, we cloned the biosensor fusion genes separately into the stable non-integrative plasmid pHT01, resulting in the fusion genes being under the control of the isopropyl β-D-1-thiogalactopyranoside (IPTG)-inducible P_*grac*_ promoter^25^,^26^ as indicated in the circuit visualised using Pigeon^16^ (Fig. 4a). Another issue that can arise when overexpressing proteins in *B. subtilis* is the degradation of such proteins by the native proteases produced by the bacterium. To reduce the native degradation of our biosensors in *B. subtilis* we used the protease deficient strain WB800N^27^ as the host.

### TEV protease whole-cell biosensors are functional and show required specificity

As a proof of principle, we wanted to show that the TEV protease-specific versions of the two biosensor designs are functional and that they show the required specificity for TEV protease. The assay used to analyse whether the biosensors can detect a specific protease is shown on the left side of Fig. 2.

For the *E. coli* housed system, all three biosensors were successfully expressed (Supplementary Fig. 1) and the proteins localised to the outer membrane in the correct orientation as evidenced by the successful labelling of the cells with streptavidin-R-phycoerythrin (SAPE) conjugate (Fig. 3b). To test if the cells expressing the fusion proteins could function as biosensors, the cells were exposed to a range of commercial proteases (AcTEV protease, Enterokinase and PreScision protease), then labelled with SAPE and the subsequent fluorescence of the cells measured using flow cytometry. The representative gating strategy is shown in Supplementary Fig. 2a. TEV (AcTEV) protease cleaved the TEV-specific mCPX-TEV biosensor (reduced to a mean % normalised fluorescence of 1% ± 0.2%) whilst the other two non-TEV motif containing sensors were not cleaved (mCPX-CON: 98.9% ± 4.9% fluorescence; mCPX-ELA: 96.6% ± 8.4% fluorescence; Fig. 3c,d). Neither Enterokinase nor PreScision protease cleaved any of the detection modules (Fig. 3c,d, Supplementary Table 2).

To establish the sensitivity of the *E. coli* whole-cell-based biosensor system, we exposed the TEV protease-specific biosensor mCPX-TEV to a range of AcTEV concentrations and assayed the cleavage of the detection module. The mCPX-TEV biosensor acted in a dose-dependent manner, with sensitivity down to 0.4 units (U) of AcTEV protease (Fig. 3e), which we estimate to be 1.5 pM of protease.

For the sensors to be housed in *B. subtilis* a main design feature was the length of the flexible linker between the anchor (LytC_CWD_) and the detection module which has the protease specific recognition motif and the epitope tag for labelling (Fig. 4a). This is due to the fact that the LytC anchor is thought to bind to teichoic acids^22^ in the cell wall layer of *B. subtilis* which is a relatively thick complex. We therefore iterated the design by using multiple linker length designs. To this end, we designed several TEV specific biosensors which had either a 5, 50 or 100 amino acid-long linker (supplementary Table 1, supplementary Fig. 3). The three linker length biosensors were expressed successfully at the correct sizes (supplementary Fig. 3c). Also, the cells were labelled, as measured by flow cytometry and the representative gating strategy shown in supplementary Fig. 2b, with the anti-6X His tag antibody Phycoerythrin conjugate (His-PE) showing the biosensors were located correctly in the cell wall (supplementary Fig. 3a,b). The design that resulted in the highest labelling of cells was the biosensor with the 100 amino acid linker (supplementary Fig. 3a,b). This was therefore the biosensor design that was used for the TEV-specific biosensor (LytC_CWD_-TEV) and also for constructing the elastase specific (LytC_CWD_-ELA) and the control (LytC_CWD_-CON) biosensors.

All three of these biosensors were expressed successfully in this host (supplementary Fig. 4) with the proteins localising to the cell wall and being exposed to the external environment, as evidenced by labelling of the cells with His-PE (Fig. 4b). To examine whether the sensors were functional when housed on the surface of *B. subtilis*, cells expressing the biosensors were exposed to the same set of commercial proteases (AcTEV protease, Enterokinase and PreScision protease) as per the *E. coli* sensors and subsequent fluorescent labelling of the cells with His-PE quantified using flow cytometry and the gating strategy shown in Supplementary Fig. 2b. TEV protease cleaved the TEV-specific biosensor LytC_CWD_-TEV (70.2% ± 4.9% fluorescence; Fig. 4c,d, supplementary Table 3) while the other two non-TEV-specific biosensors were not cleaved (LytC_CWD_-CON: 102.3% ± 3.9% fluorescence; LytC_CWD_-ELA: 100.3% ± 5.6% fluorescence). Enterokinase and PreScission protease showed no activity towards the three biosensors (Fig. 4c,d; supplementary Table 3).

To establish the sensitivity of the *B. subtilis* whole-cell-based biosensor system, cells expressing the biosensor were exposed to a range of AcTEV concentrations and the cleavage of the detection module was assayed. Similar to the *E. coli* mCPX-TEV biosensor, the *B. subtilis* LytC_CWD_-TEV biosensor acted in a dose-dependent manner, with the system able to detect 5 units (U) of AcTEV protease (Fig. 4e), which we estimate to be 18 pM of protease.

### Whole-cell biosensors can detect S. mansoni cercarial elastase

With our biosensor designs for the TEV protease working we wanted to test whether our elastase activity-specific biosensors would be able to detect their target. As mentioned earlier, our design detects the presence of *S. mansoni* via the action of the elastase protease activity they release during invasion of their primary host (Fig. 1). We obtained three biologically separate *S. mansoni* derived samples containing soluble cercarial antigens termed cercarial transformation fluid (SmCTF)^28^. The SmCTF samples were obtained by mechanical transformation of cercariae produced by the intermediate snail host *Biomphalaria glabrata*^28^ (Fig. 2).

**Figure 5:**
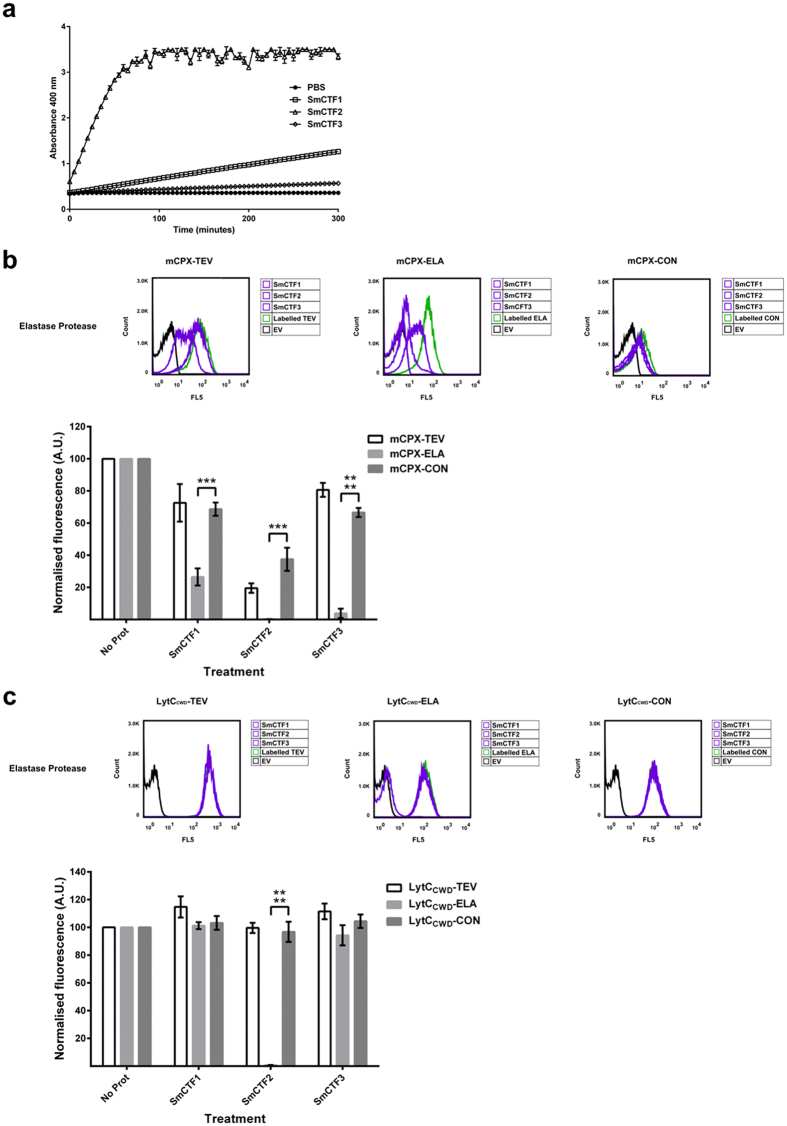
Detection of cercarial elastase activity from SmCTF biological samples. (**a**) Reconstituted SmCTF samples were incubated with either PBS (1X) or Suc-AAPF-pNA (200 mM) substrate. Proteolytic activity against Suc-AAPF-pNA altered the sample absorbance (400 nm) which was measured every 5 minutes for 5 hours. These data represent the mean absorbance ± the standard deviation of three experimental repeats. (**b**) *E. coli* expressing either mCPX-TEV, mCPX-ELA or mCPX-CON biosensors were treated with three independently generated SmCTF (1–3) biological samples. Treated cells were labelled with (SAPE)-conjugated antibody and analysed via flow cytometry with FlowJo (vX 10.0.7r2) software. A representative panel of flow cytometry data is shown and three independent biological repeats are summarised in the accompanying graph. Labelled, non-protease treated cells and *E. coli* transformed with an empty vector plasmid (EV) served as experimental controls. The fluorescence (Geometric mean FL5) of protease treated cells were normalised against labelled, non-protease treated cells (No Prot). (**c**) *B. subtilis* expressing either LytC_CWD_-TEV, LytC_CWD_-ELA or LytC_CWD_-CON biosensors were treated with three independently generated SmCTF (1–3) biological samples. Treated cells were labelled with (His-PE)-conjugated antibody and analysed via flow cytometry with FlowJo (vX 10.0.7r2) software. A representative panel of flow cytometry data is shown and three independent biological repeats are summarised in the accompanying graph. Labelled, non-protease treated cells and *B. subtilis* transformed with an empty vector plasmid (EV) served as experimental controls. The fluorescence (Geometric mean FL5) of protease treated cells were normalised against labelled, non-protease treated cells (No Prot). Student t-test ***P < 0.001 and ****P < 0.0001.

To analyse the relative amounts of cercarial elastase activity present in the three samples we decided to use a previously described assay used to detect for the activity of *S. mansoni* elastase recovered from snail shedding experiments^7^. In this assay, elastase activity was measured as activity against the substrate succinyl-ala-ala-pro-phe-*p*-nitroanilide (Suc-AAPF-pNA)^7^. Cleavage of Suc-AAPF-pNA releases 4-nitroaniline, which is yellow in colour and can be measured spectrophotometrically. Aliquots (10 μl) of SmCTF samples were incubated at 30 °C with 200 μM Suc-AAPF-pNA and the activity of cercarial elastase measured as abosorbance at 400 nm. SmCTF2 was found to have a very high enzymatic activity, whilst SmCTF1 and SmCTF3 had relatively lower activities (Fig. 5a).

We first analysed the activity of the SmCTF samples against *E. coli* cells expressing our biosensors. When the whole-cell-based biosensors were treated with the three SmCTF samples the elastase-specific sensor mCPX-ELA was cleaved (SmCTF1 26.5% ± 5.3% fluorescence, SmCTF2 0.2% ± 0.04% fluorescence, SmCTF3 3.8% ± 2.8% fluorescence; Fig. 5b, supplementary Table 2). There were some off-target effects in that the other two non-elastase sensors were, to some extent, cleaved (mCPX-CON: SmCTF1 68.7% ± 4.1% fluorescence, SmCTF2 37.5% ± 7.2% fluorescence, SmCTF3 66.6% ± 2.8% fluorescence; mCPX-TEV: SmCTF1 72.6% ± 11.7% fluorescence, SmCTF2 19.5% ± 2.9% fluorescence, SmCTF3 80.7% ± 4.3% fluorescence; Fig. 5b, supplementary Table 2) however, the elastase-specific sensor was significantly more cleaved than the other two sensors (Fig. 5b, supplementary Table 2, supplementary Table 4). The activity of the SmCTF samples towards the other two biosensors (mCPX-TEV and mCPX-CON) is, most likely, due to the presence of other proteases present in these samples, that are released from cercarial acetabular glands together with the elastase during mechanical transformation of the larvae^28^ (Fig. 2).

When the *B. subtilis* based whole-cell biosensors were exposed to the SmCTF elastase samples, the elastase-specific sensor (LytC_CWD_-ELA) was cleaved by the SmCTF2 sample (0.7% ± 0.1% fluorescence; Fig. 5c, supplementary Table 3). However, the other two SmCTF samples had little if no activity on this sensor (101.2% ± 2.5% fluorescence and 94.3% ± 7.3% fluorescence respectively; Fig. 5c, supplementary Table 3). The other two biosensors were not cleaved by the SmCTF samples (Fig. 5c, supplementary Table 3). The limited activity of SmCTF1 and SmCTF3 towards the LytC_CWD_-ELA biosensor is, most likely, due to the presence of relatively low amounts of active elastase in these samples (Fig. 5a).

### *B. subtilis* biosensors survive lyophilisation and maintain biosensor expression

**Figure 6:**
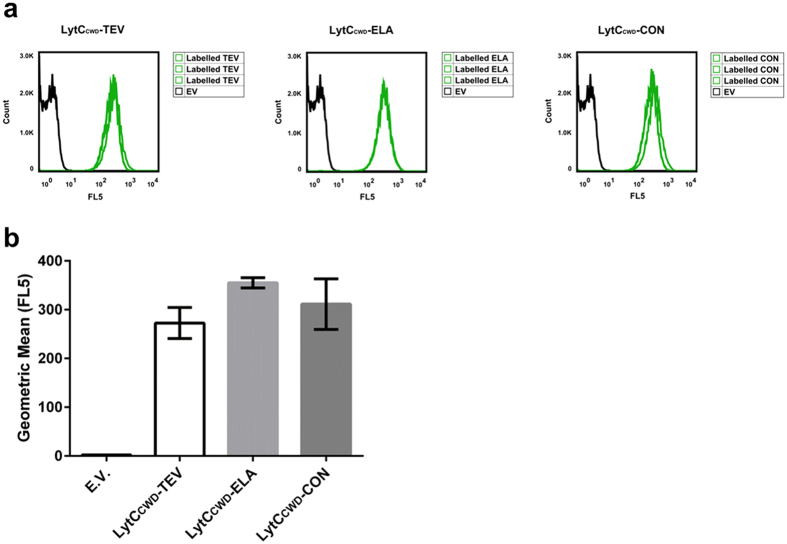
*B. subtilis* whole-cell biosensors are maintained post-lyophilisation. (**a**) Strains containing the plasmids encoding the biosensors, as well as the empty vector control, were lyophilised, reconstituted, and expression of the biosensors quantified using flow cytometry. The left hand panel, the middle panel and the right panel represent the LytC_CWD_-TEV, LytC_CWD_-ELA and LytC_CWD_-CON biosensors respectively. Empty vector (EV), Labelled TEV, Labelled ELA and Labelled CON are the untreated but labelled samples for LytC_CWD_-TEV, LytC_CWD_-ELA and LytC_CWD_-CON respectively. Flow data analysis was carried out using FlowJo (vX 10.0.7r2) software. (**b**) Summary of flow cytometry data. Mean Geometric means were: LytC_CWD_-TEV 272.7 ± 31.8, LytC_CWD_-ELA 355 ± 10.6 and LytC_CWD_-CON 311.3 ± 51.7.

To enable easy and cost effective transportation of the biosensor strains, especially in settings lacking reliable cold chain access, we wanted to show that these strains would be viable and maintain the plasmids encoding the biosensors after the process of lyophilisation. According to previous reports *B. subtilis* and related *Bacillus* strains are viable after the lyophilisation process and do maintain plasmids that they carry^29^,^30^. To test whether our host *B. subtilis* strain, WB800N, would survive the process we decided to lyophilise the strains carrying the plasmids encoding the three biosensors, as well as the empty vector control, and analyse whether the strains survive and maintain their plasmids. Cells were grown overnight, and aliquots (1 ml OD_600 nm_ 1.0) were resuspended in 50 μl fresh sterile LB medium before being lyophilised. Lyophilised cells were resuspended in sterile dH_2_O (thereby reconstituted in LB medium) and allowed to recover at room temperature for 2 hours. The cells were then added to fresh LB broth and induced to express the sensors as described in the materials and methods section. All strains survived the process and the plasmids were maintained as evidenced by the labelling of the cells with His-PE as measured by flow cytometry (Fig. 6).

## Discussion

Schistosomiasis is a neglected tropical disease (NTD), that affects millions of people and therefore the rapid and cost-effective detection of the causative agents, namely schistosomes such as *S. mansoni*, are desirable. The current gold standard diagnostic technique for schistosomiasis is the microscopic examination of excreta for the detection and identification of parasitic eggs^4^. The World Health Organisation (WHO) recommends the use and microscopic examination of polycarbonate filters for eggs in the urine, the urine haem dipstick assay^31^,^32^, or the Kato-Katz faecal examination technique^33^. Quantitative egg counts from urine and faeces are useful for epidemiological surveys as the egg load correlates well with worm burden and morbidity^4^. However, these techniques are not always sensitive, often giving false negative results from lightly infected individuals^34^. Alternative methods include serological techniques such as indirect hemagglutination (IHF) assays^35^, enzyme-linked immunosorbent assay (ELISA)^35^,^36^ to detect schistosoma-specific antibodies, or dispticks that detect schistosoma-derived antigens in the blood or urine^37^,^38^. Potential problems with using antibody-based detection techniques are that they cannot distinguish between past or present infections and may have cross-reactivity to other helminth parasites^36^,^39^,^40^. Molecular detection techniques include the Polymerase Chain Reaction (PCR)^41^,^42^, Real Time-PCR (RT-PCR)^43^,^44^,^45^, multiplex RT-PCR^46^, oligochromatographic dipsticks^45^,^47^ a Loop-mediated isothermal amplification (LAMP) assay^48^ and 16 S ribosomal RNA (rRNA) detection^49^. Additionally, other methods such as natural oil laced traps^50^ and quantifiable mouse bioassays^51^ are used *in situ* to survey for the presence of infective schistosomes. Yet in the case of the mouse bioassay there is a considerable time lag between infection and observation of possible infection.

The above techniques all require, to some degree, trained people, laboratory equipment and reagents. However, most incidences of schistosomiasis are in resource-limited settings. To this end, the development of a synthetic biology approach to systematically design, build and characterise a schistosome biosensor that is rapid, low cost and specific that can detect the parasite either *in situ* at water sites or in local laboratories near water courses is desirable. We decided to develop a modular microbial cell-based biosensor as these systems can be produced relatively cheaply and if they can be stored in lyophilised forms, can be transported without cold storage. These systems are also self-replicating and therefore provide a sustainable supply of biosensors in resource-limited settings. Furthermore, through synthetic biology, non-pathogenic bacteria and yeast have been successfully engineered from modular parts to create novel biosensors that can detect a variety of substrates at very low concentrations and with high specificity^52^. In this study we decided to implement our biosensor designs in two different hosts, *E. coli* and *B. subtilis*. *E. coli* was used to obtain proof of principle data and to develop a laboratory-based biosensor detection system for *S. mansoni*. The *E. coli* biosensors will only be used in a laboratory setting as there are some concerns about the application and release of genetically modified *E. coli* as biosensors in an environmental setting. *B. subtilis* was chosen as our *in situ* host as this is a GRAS organism. Indeed, there is precedence for using this chassis as a biosensor as it has previously been used successfully to detect for a range of environmental pollutants such as lead, cadmium and other heavy metals, as well as arsenic^53^,^54^,^55^,^56^.

In the current study, our biosensors detect the elastase activity released by cercariae on contact with human skin (Fig. 1). We chose elastase as the target for the biosensor as it is one of the invasive features of the parasite, indicating that there is an active population present and furthermore, a synthetic recognition motif for this elastase activity has been identified^7^. When both the *E. coli* and *B. subtilis-*housed biosensors were exposed to a range of different commercial proteases, the only significant responses were observed for the TEV protease specific mCPX-TEV and LytC_CWD_-TEV biosensors to AcTEV protease (Figs 3c,d and 4c,d; supplementary Tables 2–4). Furthermore, the mCPX-TEV and LytC_CWD_-TEV biosensors were able to detect relatively low amountsrof AcTEV protease, down to an estimated 1.5 pM (Fig. 3e) and 18 pM (Fig. 4e) respectively. This lower sensitivity observed for the *B. subtilis* housed TEV biosensor could be due to the biosensors being more exposed in *E. coli* compared to *B. subtilis* due to differences in their cell architectures (Figs 3a and 4a).

Having shown that the biosensor designs in both organisms are expressed and that the TEV protease-specific sensors detected TEV, we wanted to test whether the elastase-specific biosensors (mCPX-ELA and LytC_CWD_-ELA) could detect the *S. mansoni* cercarial elastase. Efforts to obtain a recombinant source of cercarial elastase have been unsuccessful, and there are no published reports of active recombinant elastase. We therefore decided to test the biosensors against elastase present in *S. mansoni* derived SmCTF preparations which contain soluble cercarial antigens^28^. These preparations are created by aspirating cercariae 15 times through a 20 gauge disposable syringe, thereby transforming the cercariae to schistosomula. As a result of the transformation the cercariae lose their tails and the cercarial glycocalyx, and the contents of the acetabular glands are released in soluble form into the superntatant thereby resulting in the SmCTF preparation^28^. Based on activity against Suc-AAPF-pNA, SmCTF2 had the highest relative elastase activity compared to SmCTF samples 1 and 3 (Fig. 5a). When the *E. coli*-housed biosensors were exposed to the three different SmCTF samples, the elastase-specific biosensor detected and was cleaved significantly more than the other two sensors (Fig. 5b; supplementary Tables 2 and 4). SmCTF2 showed the greatest ability to cleave the elastase specific biosensor mCPX-ELA, followed by SmCTF3 whilst SmCTF1 showed the weakest activity against this biosensor. This activity pattern against mCPX-ELA correlated with the relative amounts of active elastase activity present in these samples (Fig. 5a). However, we did observe some off-target responses to the three SmCTF samples in that these samples did recognise and cleave the mCPX-TEV and mCPX-CON biosensors. This could be due to the way the SmCTF samples are obtained. It has been observed previously that schistosomula have complete loss of preacetabular and postacetabular gland material^57^, and that these gland secretions are a mix of various proteins including elastase and other proteases^58^,^59^. Indeed, a metalloprotease (SmPepM8) and a dipeptidyl peptidase IV (SmDPP IV) were also identified in these secretions and are hypothesised to play a role in cercarial invasion^58^, however, we do not know if these peptidases or other gland contents would result in non-specific cleavage of our biosensors. Although, it is possible that these or other proteases that may have been released during SmCTF sample preparation are responsible for the off-target responses. When the *B. subtilis*-housed biosensors were exposed to the SmCTF samples, only the elastase specific biosensor LytC_CWD_-ELA was recognised and cleaved (Fig. 5c). However, significant cleavage only occurred when the sensor was exposed to SmCTF2, the other two SmCTF samples did not cleave the biosensor (Fig. 5c; supplementary Tables 3 and 4). This suggests that there was not a high enough concentration of elastase present in SmCTF 1 and 3 to be detected by the biosensor. What is encouraging for the *B. subtilis*-based biosensors is that the elastase present in SmCTF2 did not cause any off-target responses and cleave the non-elastase specific biosensors (Fig. 5c; supplementary Tables 3 and 4). In future studies it may be possible to use mass spectrometry and other analyses to provide insights into the composition of these secretions in order to rationally identify strategies that will result in the inhibition and/or removal of the activities of these other proteases^58^,^59^. Furthermore, these insights could aid in future iterative designs for the linkers and/or anchoring proteins that could prevent these off-target effects.

Sensitivity is an important consideration for biosensor design. However, to the best of our knowledge, no measurements of the amount of elastase released and/or produced by *S. mansoni* exist. A recent study used 3-D modelling to measure the head and gland volumes in cercariae of the related schistosoma *Trichobilharzia regenti*^60^. In that study, the volume for live anaesthetized cercariae were 700,360 μm^3^ ± 22.7% and the volumes of the preacetabular glands, which they term the circumacetabular glands, were found to total 87,545 μm^3^ ± 3.5%^60^. *T. regenti* cercariae are approximately 35% bigger in length than *S. mansoni* cercaria^60^ so if we calculate a volume based on this and the figures produced for *T. regenti* we can estimate that the volume of *S. mansoni* is approximately 455,234 μm^3^. Furthermore, if we make the assumption that the glands are 35% bigger in size in *T. regenti* compared to those of *S. mansoni* this gives the preacetabular glands of the latter an approximate volume of 56,904 μm^3^. The preacetabular glands are responsible for secreting elastase^61^. Based on the estimated size of the glands and, along with reports that cercariae produce and store elastase in readiness for invasion, we can assume that cercarial larva each produce elastase in the pg range. In order to detect elastase in this range, we are currently investigating the possibility of using a membrane coated in linoleic acid to attract cercariae and with which they can make contact thereby encouraging release of the elastase from their glands. These membranes could conceivably be encased in a trap and placed in a freshwater body that is suspected of harbouring the parasite. Then water samples taken from the trap could be applied to our biosensors for detection.

We wanted to make our *in situ* biosensor design easy and cost effective to transport and store especially in areas lacking a reliable access to a cold chain. Previous studies have shown that *Bacillus* strains are viable after the lyophilisation process and maintain plasmids they harbour^29^,^30^. Importantly, our strains both survived and maintained the biosensor encoding plasmids (Fig. 6). This allows the biosensors to be transported in a lyophilised form in a cost-effective way and will facilitate their use at *in situ* test sites. Indeed, this will also open up the possibility of using our biosensors in epidemiological studies to map the populations of *S. mansoni* to observe any spread of the populations or re-emergence of populations in areas declared free of infection^62^.

To our knowledge, these are the first whole-cell-based biosensors that have been directly applied to the detection of *S. mansoni* and they offer the possibility of developing further whole-cell-based biosensors for other parasites.

## Methods

### Bacterial strains, plasmids and growth conditions

All strains used in this study are listed in Supplementary Table 5. *Escherichia coli* and *Bacillus subtilis* strains were grown in Luria-Bertani (LB) medium at 37 °C (*E. coli*) or 30 °C (*B. subtilis*) unless otherwise stated. When applicable, the medium was supplemented with the following antibiotics: *E. coli* cultures – ampicillin (Amp) 100 μg/ml; chloramphenicol (Cam) 50 μg/ml; kanamycin (Kan) 35 μg/ml; *B. subtilis* cultures – chloramphenicol (Cam) 5 μg/ml; Kanamycin (Kan) 10 μg/ml. It should be noted that Kan is used to select for the Neomycin (NeoR) resistance gene in *B. subtilis*.

### Strain and plasmid construction

Oligonucleotide primers used for plasmid construction and sequencing are listed in Supplemental Table 6.

#### E. coli biosensor constructs and strains

The prototype mCPX-TEV sensor device consisting of the *xylF* promoter (BBa_I741018), B0034 RBS (BBa_B0034) and *mCPX-TEV* gene cloned into the plasmid pSB3C5 (strain pNK5) was transformed into *E. coli* strain NEB10-beta, resulting in the construction of strain pAJW13. The elastase-specific sensor was constructed via inverted PCR using plasmid pNK5 (pSB3C5-*xylF*-B0034-*mCPX-TEV*) as a template and with the primer pair 64/65, the resultant DNA product purified, phosphorylated and self-ligated resulting in the construction of plasmid pAJW14 (pSB3C5-*xylF*-B0034-*mCPX-ELA*). To counteract any deleterious effects due to overexpression of the sensors, the B0034 RBS was replaced with the B0032 RBS (BBa_B0032) via inverse PCR using primer pair 66/67 and plasmids pNK5 and pAJW14 as templates. The DNA products were then purified, phosphorylated and self-ligated, resulting in plasmids pSB3C5-*xylF*-B0032-*mCPX-TEV* (pAJW15) and pSB3C5-*xylF*-B0032-*mCPX-ELA* (pAJW16) respectively. The control biosensor was constructed via inverted PCR using primer pair 180/181 and plasmid pAJW15 as the template, the DNA product purified, phosphorylated and self-ligated resulting in the construction of plasmid pSB3C5-*xylF*-B0032-*mCPX-CON* (pAJW66). Plasmid pSB3C5-*xylF* (pNK4) was transformed into *E. coli* NEB10-beta, resulting in strain pAJW12 and acts as the empty vector control.

#### B. subtilis biosensor constructs and strains

The fusion gene containing the cell wall-binding domain of *B. subtilis lytC* (*lytC*_*CWD*_) with a five amino acid linker and the TEV protease detection module was synthesised and cloned into pMK-RQ by GeneArt (Life Technologies, UK), and the resulting plasmid was transformed into *E. coli* NEB10-beta (pAJW17). The cell wall-binding domain of *lytC* possesses a native *Acc*I restriction site which was utilised for some subsequent cloning steps. Fusion genes containing a small fraction of *lytC*_*CWD*_ starting with the native *Acc*I restriction site with either a fifty or a one hundred amino acid linker and the TEV detection module were synthesised and cloned into pMA-T by GeneArt (Life Technologies, UK), and the resulting plasmids transformed into *E. coli* NEB10-beta to give plasmids pAJW29 and pAJW47 respectively. To construct the full length TEV protease sensors with either a fifty amino acid linker (*lytC*_*CWD*_*-50AA-TEV*) or a one hundred amino acid linker (*lytC*_*CWD*_*-100AA-TEV*), plasmids pAJW29 and pAJW47 were digested with enzymes *Acc*I and *Spe*I and the *lytC*_*CWD*_*-50AA-TEV* and *lytC*_*CWD*_*-100AA-TEV* fragments ligated into plasmid pAJW17, which had been cut with the same enzymes, thereby replacing the *lytC*_*CWD*_*-5AA-TEV* fragment. The resultant plasmids, pMK-RQ-*lytC*_*CWD*_*-50AA-TEV* (pAJW30) and pMK-RQ-*lytC*_*CWD*_*-100AA-TEV* (pAJW52) were transformed into *E. coli* NEB10-beta. The elastase-specific and control sensors were constructed as follows: inverted PCR using primer pairs 148/149 and 291/292 and plasmid pAJW47 as a template were used to replace the TEV specific recognition motif with that for elastase or the control biosensor respectively. DNA products were purified, phosphorylated, self-ligated and transformed into *E. coli* NEB10-beta, resulting in the plasmids pMA-T-*100AA-ELA* (pAJW67) and pMA-T-*100AA-CON* (pAJW105). To construct the full length elastase and control sensors, plasmids pAJW67 and pAJW105 were digested with enzymes *Acc*I and *Spe*I and the *lytC*_*CWD*_*-100 A-ELA* and *lytC*_*CWD*_*-100 A-CON* fragments ligated into plasmid pAJW17, which had been cut with the same enzymes, replacing the *lytC*_*CWD*_*-5AA-TEV* fragment. The resulting plasmids, pMK-RQ-*lytC*_*CWD*_*-100AA-ELA* (pAJW68) and pMK-RQ-*lytC*_*CWD*_*-100AA-CON* (pAJW106) were transformed into *E. coli* NEB10-beta.

For construction of plasmids to introduce the sensors into *B. subtilis* WB800N (MoBiTech GmbH, Germany), the biosensor fusion genes were cloned independently into the non-integrative *Bacillus* expression vector pHT01 (MoBiTech GmbH, Germany). The sensors *lytC*_*CWD*_*-5AA-TEV*, *lytC*_*CWD*_*-50AA-TEV*, *lytC*_*CWD*_*-100AA-TEV*, *lytC*_*CWD*_*-100AA-ELA*, and *lytC*_*CWD*_*-100AA-CON* were amplified using primer pair 70/150 and plasmids pAJW17, pAJW30, pAJW52, pAJW68 and pAJW106 as the templates respectively. The PCR products were subsequently digested with enzymes *Bam*HI and *Xba*I and ligated with vector pHT01, which had been digested with the same enzymes, resulting in the construction of plasmids pHT01- *lytC*_*CWD*_*-5AA-TEV* (pAJW21), pHT01-*lytC*_*CWD*_*-50AA-TEV* (pAJW35), pHT01- *lytC*_*CWD*_*-100AA-TEV* (pAJW57), pHT01-*lytC*_*CWD*_*-100AA-ELA* (pAJW71) and pHT01-*lytC*_*CWD*_*-100AA-CON* (pAJW110). The plasmids, as well as the pHT01 empty vector, were transformed into *B. subtilis* WB800N using the two-step transformation procedure as described previously^63^ and transformants were selected on LB agar containing the appropriate antibiotics. This resulted in strains WB800N pHT01 (AJW5), WB800N pHT01-*lytC*_*CWD*_*-5AA-TEV* (AJW6), WB800N pHT01-*lytC*_*CWD*_*-50AA-TEV* (AJW10), WB800N pHT01-*lytC*_*CWD*_*-100AA-TEV* (AJW15), WB800N pHT01-*lytC*_*CWD*_*-100AA-ELA* (AJW22) and WB800N pHT01-*lytC*_*CWD*_*-100AA-CON* (AJW23).

The DNA sequences of all inserts/constructs were verified by the Sanger sequencing service provided by Source BioScience (Cambridge, UK). Primers AJW10 and AJW11 were used to sequence pSB3C5 based constructs, primers AJW80 and AJW81 were used to sequence pMA-T and pMK-RQ based constructs, and primers AJW77 and AJW78 were used to sequence pHT01 based constructs. Primers AJW73 – AJW76 were used to sequence the *lytC* fusion constructs.

### Induction of Biosensor expression

#### E. coli biosensor strains

Single colonies were used to inoculate LB medium and cultures were grown at 37 °C with shaking at 220 rpm. Over-night cultures were subcultured into fresh LB medium (1:20) and grown at 37 °C with shaking at 220 rpm, until cultures reached an OD_600 nm_ of between 0.5 and 0.6. Cultures were then split into two sets: uninduced and induced with either 0 or 100 mM xylose. Cultures were then incubated over-night at 30 °C with shaking at 220 rpm.

#### *B. subtilis* biosensor strains

Single colonies were used to inoculate LB medium and cultures were grown at 30 °C with shaking at 180 rpm. Over-night cultures were subcultured into fresh LB medium to an OD_600 nm_ of 0.15 and grown at 30 °C with shaking at 180 rpm, until cultures reached an OD_600 nm_ of between 0.7 and 0.8. Cultures were then split into two sets: uninduced and induced with either 0 or 1 mM IPTG. Cultures were then incubated over-night at 30 °C with shaking at 180 rpm.

### Biosensor detection by Western blot

Biosensor protein detection by Western blot was undertaken as follows. Briefly, for sodium dodecyl sulphate (SDS)-polyacrylamide gel electrophoresis (PAGE) and Western blot analysis of His-tagged biosensor proteins, 1 ml of OD_600 nm_ 1.0 overnight culture was centrifuged at 12,470 *g* for 2 min. The resultant pellet was washed with 1 ml sterile phosphate buffered saline (PBS, 1 X) and centrifuged again at 12,470 *g.* Cell pellets were then resuspended in 50 μl protein sample buffer containing 2% SDS. Samples were boiled for 10 min and 10 μl of samples were loaded onto 12% SDS-PAA gels. Samples were probed with HRP-conjugated His-tag-specific monoclonal antibody (A7058, Sigma-Aldrich, UK) used at a 1:4,000 dilution and Western blots were developed by enhanced chemiluminescence (ECL). Westerns blots were performed on at least 3 independently grown cultures.

### Whole-cell labelling with fluorescent probes

Cells were labelled with fluorescent probes as follows. Briefly, 1 ml of OD_600 nm_ 1.0 cultures were centrifuged at 12,470 *g* for 2 min and the supernatant aspirated. Cells were washed with 1 ml sterile PBS (1 X) and pelleted again by centrifugation at 12,470 *g* for 2 min. The supernatant was aspirated and cultures resuspended in 50 μl PBS (1 X). *E. coli* cultures were then labelled by the addition of 1.25 μg SAPE (SNN1007, Life technologies, UK), whilst *B. subtilis* cultures were labelled by the addition of 2.5 μg His-PE (ab72467, Abcam, UK). Cultures were mixed briefly by vortexing and incubated at room temperature for 30 min. After incubation, samples were centrifuged at 12,470 *g* for 2 min, the supernatant aspirated and the cell washed with 100 μl PBS (1 X). The cells were centrifuged once more at 12,470 *g*, the supernatant aspirated and the cells resuspended in 100 μl PBS (1 X) and kept on ice until required.

### Preparation of SmCTF samples

Three *S. mansoni* SmCTF samples were produced by BioGlab Ltd. (Nottingham, UK) as described previously^28^. Freeze-dried aliquots were reconstituted in sterile distilled water and stored at −20 °C until use.

### Biosensor assays

Protease cleavage assays on cells expressing the biosensors were undertaken as follows. Briefly, 1 ml (OD_600 nm_ 1.0) aliquots of induced cells were centrifuged at 12,470 *g*, the supernatant aspirated and the cells washed in 1 ml PBS (1 X). The cells were then centrifuged again at 12,470 *g*, the supernatant aspirated and cell pellets resuspended with PBS (1 X) to a volume specific to the particular cleavage assay, up to a maximum reaction volume of 100 μl. TEV (AcTEV) protease cleavage assay: cells were resuspended in 93 μl PBS (1 X), 5 μl 20 X TEV buffer (1 M Tris-HCl pH 8.0, 10 mM EDTA), 1 μl DTT (0.1 M) and 1 μl AcTEV (10 U; 12575-015, Life Technologies, UK) was added. Recombinant Enterokinase (rEK) cleavage assay: cells were resuspended in 89 μl PBS (1 X), 10 μl 10 X rEK cleavage buffer (500 mM NaCL, 200 mM Tris-HCl, 20 mM CaCl_2_, pH 7.4) and 1 μl rEK (1 U; 69066-3, Novagen, UK) was added. PreScission protease cleavage assay: cells were resuspended in 99 μl PBS (1 X) and 1 μl of PreScission protease (2 U; 27-0843-01, GE Healthcare Life Sciences, UK) was added. SmCTF elastase cleavage assay: cells were resuspended in 90 μl PBS (1 X) and 10 μl of relevant SmCTF sample added. For the mCPX-TEV and LytC_CWD_-TEV sensitivity assays the extra volumes of enzymes required replaced the same volume of PBS (1 X). Relevant buffer controls for the AcTEV and recombinant Enterokinase reactions replaced the volume of enzyme added with the same volume of PBS (1 X). Control samples comprised cells resuspended in 100 μl PBS (1 X). Samples were then mixed briefly by vortexing and incubated statically at 30 °C for 1 hour. After incubation, samples were centrifuged at 12,470 *g* for 2 min, the supernatant aspirated and the cells washed in 100 μl PBS (1 X). Cells were then centrifuged again at 12,470 *g* for 2 min, the supernatant aspirated and the cells resuspended in 50 μl PBS (1 X) and stored on ice until labelled with SAPE (*E. coli* cells) or His-PE (*B. subtilis* cells).

### Flow cytometry

Labelled cell samples were diluted (1:1,000) into PBS (1 X) and loaded onto a BD-FACScan flow cytometer (Becton Dickinson, UK) for detection of streptavidin-R-phycoerythrin (SAPE) conjugate labelling of *E. coli* samples or His-Phycoerythrin (His-PE) conjugate labelling of *B. subtilis* samples. At least 45,000 cells per sample were measured (SAPE/ His-PE - FL5 detector, Excitation 561 nm Emission 612/25 nm) and data analysis from three biological replicates was carried out using FlowJo (vX 10.0.7r2) software (FlowJo, LLC, Oregon, USA). For biosensor assay data, the background signal, as determined by the average geometric mean (FL5) of labelled empty vector cells was removed. Subsequently, these data were normalised to the average geometric mean (FL5) of the corresponding labelled, non-protease treated biosensor control. For the SmCTF and PreScission protease treatments, fluorescence was normalised to the relevant non-protease non-treatment control, TEV treated cells were normalised to TEV buffer control and the Enterokinase treated cells were normalised to the Enterkinase buffer control. Treatments were normalised to controls from the same biological set. In regards to the data generated for the linker length study and the effect of lyophilisation on biosensor maintenance, the data presented are the mean geometric means (FL5) of the samples.

### SmCTF elastase activity against N-succinyl-ala-ala-pro-phe-p-nitroanilide

The activity of the SmCTF samples was assayed in triplicate as previously described^7^. Briefly, 10 μl aliquots of SmCTF samples, or 10 μl of PBS (1 X; negative control) were assayed in 100 μl of reaction buffer (100 mM glycine, pH 9.0, 200 μM N-succinyl-ala-ala-pro-phe *p*-nitroanilide (Suc-AAPF-pNA); Sigma-Aldrich, UK) at 30 °C. Fluorescence was measured every 5 minutes at 400 nm, with 10 seconds of shaking at 400 rpm before each measurement in a BMG Clariostar plate reader (BMG, UK).

### Lyophilisation of biosensor expression strains

Lyophilisation and subsequent revival of biosensor expressing *B. subtilis* strains was undertaken as follows. Cells containing plasmids encoding the biosensors or the empty vector control were grown overnight in LB medium with appropriate antimicrobials at 30 °C with shaking at 180 rpm. Aliquots (1 ml OD_600 nm_ 1.0) of the cultures were taken, centrifuged at 12 470 *g* for 2 min and the supernatant aspirated. The cells were resuspended in 50 μl of sterile LB medium and then lyophilised for 90 min using the ScanVac CoolSafe freeze dryer (LaboGene, Lynge, Denmark). After lyophilisation, the cells were resuspended in 50 μl sterile dH_2_0 (thus reconstituted in LB medium), left for 2 hours at room temperature to reconstitute, and then added to 5 ml fresh LB medium with appropriate antimicrobials. The cultures were incubated overnight at 30 °C with shaking at 180 rpm. These cultures were then subcultured and induced to express the biosensors as described above. Cells expressing the biosensors were labelled with His-PE and quantified using flow cytometry as described above.

### Statistics

Statistical analysis (standard deviation and unpaired t-test) was carried out on at least three experimental replicates using GraphPad Prism 6.05 (GraphPad Software Inc., La Jolla, California).

## Additional Information

**How to cite this article**: Webb, A. J. *et al.* A protease-based biosensor for the detection of schistosome cercariae. *Sci. Rep.* **6**, 24725; doi: 10.1038/srep24725 (2016).

## Supplementary Material

Supplementary Information
