# Supplementary material for: A protease-based biosensor for the detection of schistosome cercariae
Source: Sci Rep. 2016 Apr 19;6:24725. doi: 10.1038/srep24725 (PMC4835807; doi:10.1038/srep24725)
Supplement: Supplementary Information [file srep24725-s1.pdf]

Supplementary files.

A protease-based biosensor for the detection of schistosome cercariae

A. J. Webb[1,2], R. Kelwick[1,2], M. J. Doenhoff[3], N. Kylilis[1,2], J. T. MacDonald[1,2], K. Y. Wen[1,2], C. McKeown[2], G. Baldwin[1,4], T. Ellis[1, 5], K. Jensen[1,2] & P. S. Freemont[1,2*]

[1]Centre for Synthetic Biology and Innovation, Imperial College London, London, UK, [2] Section of Structural Biology, Department of Medicine, Imperial College London, London, UK, [3]School of Life Sciences, University of Nottingham, Nottingham, UK, [4]Department of Life Sciences, Imperial College London, London, UK, [5]Department of Bioengineering, Imperial College London, London, UK.

*Correspondence address:

Professor Paul S. Freemont
Centre for Synthetic Biology and Innovation
Department of Medicine
Sir Ernst Chain Building
South Kensington Campus
Exhibition Road
London
SW7 2AZ
UK
Email: p.freemont@imperial.ac.uk
Tel: +44 (0) 207 594 5327

Supplementary Table 1. Protein sequences of *E. coli* and *B. subtilis* biosensors

Sensor	Notes	Sequence
E. coli sensors		
mCPX-TEV	OmpX signal peptide:yellow; Stretavidin-binding peptide: green; flexible linkers: blue; TEV recognition motif: red; CPX: pink; His tag: grey.	MKKIACLSALAAVLAFTAGTSVAWCHPMWEVMCLRGQSGQENL<br>YFQGGGQSGQSGDYNKNQYYGITAGPAYRINDWASIYGVVGVGY<br>GKFQTTEYPTYKHDTSDYGFSYGAGLQFNPMENVALDFSYEQSRI<br>RSVDVGTWIAGVGYRFGGSGATSTVTGGYAQSDAQGQMNKMGG<br>FNLKYRYEEDNSPLGVIGSFTYTEKSRTASHHHHHH
mCPX-ELA	OmpX signal peptide:yellow; Stretavidin-binding peptide: green; flexible linkers: blue; Elastase recognition motif: red; CPX: pink; His tag: grey.	MKKIACLSALAAVLAFTAGTSVAWCHPMWEVMCLRGQSGQSWP<br>LGGQSGQSGDYNKNQYYGITAGPAYRINDWASIYGVVGVGYGKF<br>QTTEYPTYKHDTSDYGFSYGAGLQFNPMENVALDFSYEQSRIRSV<br>DVGTWIAGVGYRFGGSGATSTVTGGYAQSDAQGQMNKMGGFNL<br>KYRYEEDNSPLGVIGSFTYTEKSRTASHHHHHH
mCPX-CON	OmpX signal peptide:yellow; Stretavidin-binding peptide: green; flexible linkers: blue; Control motif: red; CPX: pink; His tag: grey.	MKKIACLSALAAVLAFTAGTSVAWCHPMWEVMCLRGQSGQGSS<br>QSGGGQSGQSGDYNKNQYYGITAGPAYRINDWASIYGVVGVGYG<br>KFQTTEYPTYKHDTSDYGFSYGAGLQFNPMENVALDFSYEQSRIR<br>SVDVGTWIAGVGYRFGGSGATSTVTGGYAQSDAQGQMNKMGGF<br>NLKYRYEEDNSPLGVIGSFTYTEKSRTASHHHHHH
B. subtilis sensors		
LytC$_{CWD}$-TEV (5aa)	LytC$_{CWD}$: green; flexible linkers: yellow; TEV recognition motif: red; His tag: grey.	MRSYIKVLTMCFLGLILFVPTALADNSVKRVGGSNRYGTAVQISK<br>QMYSTASTAVIVGGSSYADAISAAPLAYQKNAPLLYTNSDKLSYE<br>TKTRLKEMQTKNVIIVGGTPAVSSNTANQIKSLGISIKRIAGSNRYD<br>TAARVAKAMGATSKAVILNGFLYADAPAVIPYAAKNGYPILFTNK<br>TSINSATTSVIKDKGISSTVVVGGTGSISNTVYNKLPSPTRISGSNRY<br>ELAANIVQKLNLSTSTVYYVSNGFSYPDSIAGATLAAKKKQSLILTN<br>GENLSTGARKIIGSKNMSNFMIIGNTPAVSTKVANQLKNPGQSGQE<br>NLYFQGGGQSGQHHHHHH
LytC$_{CWD}$-TEV (50aa)	LytC$_{CWD}$: green; flexible linkers: yellow; TEV recognition motif: red; His tag: grey.	MRSYIKVLTMCFLGLILFVPTALADNSVKRVGGSNRYGTAVQISK<br>QMYSTASTAVIVGGSSYADAISAAPLAYQKNAPLLYTNSDKLSYE<br>TKTRLKEMQTKNVIIVGGTPAVSSNTANQIKSLGISIKRIAGSNRYD<br>TAARVAKAMGATSKAVILNGFLYADAPAVIPYAAKNGYPILFTNK<br>TSINSATTSVIKDKGISSTVVVGGTGSISNTVYNKLPSPTRISGSNRY<br>ELAANIVQKLNLSTSTVYYVSNGFSYPDSIAGATLAAKKKQSLILTN<br>GENLSTGARKIIGSKNMSNFMIIGNTPAVSTKVANQLKNPGQQSGG<br>SSQGGSGSSSGGQGGGSQQSSSGGSQQSQQSQGSGQQSGQQGSQE<br>NLYFQGGGQSGQHHHHHH
LytC$_{CWD}$-TEV (100aa)	LytC$_{CWD}$: green; flexible linkers: yellow; TEV recognition motif: red; His tag: grey.	MRSYIKVLTMCFLGLILFVPTALADNSVKRVGGSNRYGTAVQISK<br>QMYSTASTAVIVGGSSYADAISAAPLAYQKNAPLLYTNSDKLSYE<br>TKTRLKEMQTKNVIIVGGTPAVSSNTANQIKSLGISIKRIAGSNRYD<br>TAARVAKAMGATSKAVILNGFLYADAPAVIPYAAKNGYPILFTNK<br>TSINSATTSVIKDKGISSTVVVGGTGSISNTVYNKLPSPTRISGSNRY<br>ELAANIVQKLNLSTSTVYYVSNGFSYPDSIAGATLAAKKKQSLILTN<br>GENLSTGARKIIGSKNMSNFMIIGNTPAVSTKVANQLKNPGQQSGG<br>SSQGGSGSSSGGQGGGSQQSSSGGSQQSQQSQGSGQQSGQQGSQG<br>QQSGGSSQGGSGSSSGGQGGGSQQSSSGGSQQSQQSQGSGQQSGQ<br>QGSQENLYFQGGGQSGQHHHHHH
LytC$_{CWD}$-ELA (100aa)	LytC$_{CWD}$: green; flexible linkers: yellow; Elastase recognition motif: red; His tag: grey.	MRSYIKVLTMCFLGLILFVPTALADNSVKRVGGSNRYGTAVQISK<br>QMYSTASTAVIVGGSSYADAISAAPLAYQKNAPLLYTNSDKLSYE<br>TKTRLKEMQTKNVIIVGGTPAVSSNTANQIKSLGISIKRIAGSNRYD<br>TAARVAKAMGATSKAVILNGFLYADAPAVIPYAAKNGYPILFTNK<br>TSINSATTSVIKDKGISSTVVVGGTGSISNTVYNKLPSPTRISGSNRY<br>ELAANIVQKLNLSTSTVYYVSNGFSYPDSIAGATLAAKKKQSLILTN<br>GENLSTGARKIIGSKNMSNFMIIGNTPAVSTKVANQLKNPGQQSGG<br>SSQGGSGSSSGGQGGGSQQSSSGGSQQSQQSQGSGQQSGQQGSQG<br>QQSGGSSQGGSGSSSGGQGGGSQQSSSGGSQQSQQSQGSGQQSGQ<br>QGSQSWPLGGQSGQHHHHHH
LytC$_{CWD}$-CON (100aa)	LytC$_{CWD}$: green; flexible linkers: yellow; Control motif: red; His tag: grey.	MRSYIKVLTMCFLGLILFVPTALADNSVKRVGGSNRYGTAVQISK<br>QMYSTASTAVIVGGSSYADAISAAPLAYQKNAPLLYTNSDKLSYE<br>TKTRLKEMQTKNVIIVGGTPAVSSNTANQIKSLGISIKRIAGSNRYD<br>TAARVAKAMGATSKAVILNGFLYADAPAVIPYAAKNGYPILFTNK<br>TSINSATTSVIKDKGISSTVVVGGTGSISNTVYNKLPSPTRISGSNRY<br>ELAANIVQKLNLSTSTVYYVSNGFSYPDSIAGATLAAKKKQSLILTN<br>GENLSTGARKIIGSKNMSNFMIIGNTPAVSTKVANQLKNPGQQSGG<br>SSQGGSGSSSGGQGGGSQQSSSGGSQQSQQSQGSGQQSGQQGSQG<br>QQSGGSSQGGSGSSSGGQGGGSQQSSSGGSQQSQQSQGSGQQSGQ<br>QGSQGSSQSGGGQSGQHHHHHH

The putative signal sequence cleavage site for LytC$_{CWD}$[1] is underlined

Supplementary Table 2. Percentage of *E. coli* biosensor cells labelled after various protease treatments

Treatment	Cell-based biosensor labelling (%)[a]		
	mCPX-TEV	mCPX-ELA	mCPX-CON
**Labelled**	100	100	100
**TEV**	1.0 ± 0.17	96.61 ± 8.36	98.92 ± 4.87
**Enterokinase**	99.92 ± 2.41	94.78 ± 2.19	102.63 ± 4.46
**PreScission**	110.0 ± 10.06	108.92 ± 9.84	96.76 ± 1.33
**SmCTF1**	72.57 ± 11.69	26.46 ± 5.31	68.67 ± 4.1
**SmCTF2**	19.53 ± 2.95	0.18 ± 0.04	37.5 ± 7.21
**SmCTF3**	80.66 ± 4.33	3.82 ± 2.89	66.58 ± 2.81

[a]Percentage fluorescence is calculated as a normalised percentage of the fluorescence of the relevant non-protease, non-treated control from the same biological set. The percentages were calculated from three independent experiments with the standard deviation of the mean shown.

Supplementary Table 3. Percentage of *B. subtilis* cells labelled after various protease treatments

Treatment	Cell-based biosensor labelling (%)[a]		
	LytC$_{CWD}$-TEV	LytC$_{CWD}$-ELA	LytC$_{CWD}$-CON
**Labelled**	100	100	100
**TEV**	70.24 ± 4.92	100.34 ± 5.63	102.34 ± 3.95
**Enterokinase**	108.04 ± 4.32	109.32 ± 6.53	98.91 ± 12.93
**PreScission**	108.27 ± 9.74	105.27 ± 4.94	103.94 ± 3.45
**SmCTF1**	114.75 ± 7.64	101.24 ± 2.52	103.23 ± 4.96
**SmCTF2**	99.62 ± 3.69	0.68 ± 0.11	96.77 ± 7.29
**SmCTF3**	111.49 ± 5.64	94.3 ± 7.26	104.43 ± 4.83

[a]Percentage fluorescence is calculated as a normalised percentage of the fluorescence of the relevant non-protease, non-treated control from the same biological set. The percentages were calculated from three independent experiments with the standard deviation of the mean shown.

Supplementary Table 4. Statistical analysis of responses of both *E. coli* and *B. subtilis* biosensors

**Comparison**[a]	**Treatment**[b]	**P-value**	**Significantly different**
**mCPX-CON vs mCPX-TEV**	TEV	<0.0001	Yes, ****
**mCPX-TEV vs mCPX-ELA**	TEV	<0.0001	Yes, ****
**mCPX-CON vs mCPX-ELA**	TEV	0.7001	No
**mCPX-CON vs mCPX-ELA**	SmCTF1	0.0004	Yes, ***
**mCPX-TEV vs mCPX-ELA**	SmCTF1	0.0034	Yes, **
**mCPX-CON vs mCPX-TEV**	SmCTF1	0.6143	No
**mCPX-CON vs mCPX-ELA**	SmCTF2	0.0009	Yes, ***
**mCPX-TEV vs mCPX-ELA**	SmCTF2	0.0003	Yes, ***
**mCPX-CON vs mCPX-TEV**	SmCTF2	0.0162	Yes, *
**mCPX-CON vs mCPX-ELA**	SmCTF3	<0.0001	Yes, ****
**mCPX-TEV vs mCPX-ELA**	SmCTF3	<0.0001	Yes, ****
**mCPX-CON vs mCPX-TEV**	SmCTF3	0.0091	Yes, **
**$LytC_{CWD}$-CON vs $LytC_{CWD}$-TEV**	TEV	0.0009	Yes, ***
**$LytC_{CWD}$-TEV vs $LytC_{CWD}$-ELA**	TEV	0.0022	Yes, **
**$LytC_{CWD}$-CON vs $LytC_{CWD}$-ELA**	TEV	0.6404	No
**$LytC_{CWD}$-CON vs $LytC_{CWD}$-ELA**	SmCTF1	0.5693	No
**$LytC_{CWD}$-TEV vs $LytC_{CWD}$-ELA**	SmCTF1	0.0437	Yes, *
**$LytC_{CWD}$-CON vs $LytC_{CWD}$-TEV**	SmCTF1	0.0935	No
**$LytC_{CWD}$-CON vs $LytC_{CWD}$-ELA**	SmCTF2	<0.0001	Yes, ****
**$LytC_{CWD}$-TEV vs $LytC_{CWD}$-ELA**	SmCTF2	<0.0001	Yes, ****
**$LytC_{CWD}$-CON vs $LytC_{CWD}$-TEV**	SmCTF2	0.5783	No
**$LytC_{CWD}$-CON vs $LytC_{CWD}$-ELA**	SmCTF3	0.1147	No
**$LytC_{CWD}$-TEV vs $LytC_{CWD}$-ELA**	SmCTF3	0.0317	Yes, *
**$LytC_{CWD}$-CON vs $LytC_{CWD}$-TEV**	SmCTF3	0.1750	No

Student t-test, *P<0.05, **P<0.01, ***P<0.001 and ****P<0.0001.

[a]The two biosensors analysed against each other

[b]The protease treatment the sensors being compared were exposed to

Supplementary Table 5. Bacterial strains and plasmids used in this study

Strain	Relevant features	Reference
***Escherichia coli* strains**		
NEB10-beta	Δ(*ara -leu*) 7697 *araD139 fhuA* Δ*lacX74 galK16 galE15 e14-* ϕ*80*d*lacZ*Δ*M15 recA1 relA1endA1 nupG rpsL* (StrR) *rph spoT1* Δ(*mrr-hsdRMS-mcrBC*); Cloning strain and host for biosensors expression	New England Biolabs
TOP10	F- *mcrA* Δ(*mrr-hsd*RMS-*mcr*BC) ϕ 80*lac*ZΔM15 Δ*lac*X74 *rec*A1 *ara*D139 Δ(*ara-leu*)7697 *gal*U *gal*K *rps*L(StrR) *end*A1 *nup*G; Cloning strain	Life Technologies
pNK4	TOP10 pSB3C5-*xylF*; *xylF* promoter BBa_I741018; CamR	This study
pNK5	TOP10 pSB3C5-*xylF*-B0034-*mCPX-TEV*; TEV protease sensor consisting of *xylF* promoter, B0034 RBS and *mCPX-TEV*; CamR	This study
pAJW9	NEB10-beta pHT01; *E. coli*/*B. subtilis* shuttle vector; contains IPTG inducible P*grac* promoter for expression of inserts in *B. subtilis*; CamR, AmpR	MoBiTec GmbH; [2]
pAJW12	NEB10-beta pSB3C5-*xylF*; CamR	This study
pAJW13	NEB10-beta pSB3C5-*xylF*-B0034-*mCPX-TEV*; plasmid pNK5 in strain NEB10-beta	This study
pAJW14	NEB10-beta pSB3C5-*xylF*-B0034-*mCPX-ELA*; Elastase sensor consisting of *xylF* promoter, B0034 RBS and *mCPX-ELA*; CamR	This study
pAJW15	NEB10-beta pSB3C5-*xylF*-B0032-*mCPX-TEV*; TEV protease sensor consisting of *xylF* promoter, B0032 RBS and *mCPX-TEV*; CamR	This study
pAJW16	NEB10-beta pSB3C5-xylF-B0032-*mCPX-ELA*; Elastase sensor consisting of *xylF* promoter, B0032 RBS and *mCPX-ELA*; CamR	This study
pAJW17	NEB10-beta pMK-RQ-*lytC*$_{CWD}$*-5AA-TEV*; fusion gene containing the cell wall binding domain of *B. subtilis lytC* with a 5 amino acid linker and the TEV detection module; KanR	GeneArt/This study
pAJW21	NEB10-beta pHT01-*lytC*$_{CWD}$*-5AA-TEV*; *lytC*$_{CWD}$*-5AA-TEV* from pAJW17 cloned into the *Bam*HI/*Xba*I sites of pHT01; AmpR, CamR	This study
pAJW29	NEB10-beta pMA-T-*50AA-TEV*; fusion gene containing a small fraction of the cell wall binding domain of *B. subtilis lytC*, a 50 amino acid linker and the TEV detection module; AmpR	GeneArt/This study
pAJW30	NEB10-beta pMK-RQ-*lytC*$_{CWD}$*-50AA-TEV*; *Acc*I/*Spe*I fragment containing *50AA-TEV* from pAJW29 cloned into the same sites of pAJW17, replacing *5AA-TEV* with *50AA-TEV*; KanR	This study
pAJW35	NEB10-beta pHT01-*lytC*$_{CWD}$*-50AA-TEV*; *lytC*$_{CWD}$*-50AA-TEV* from pAJW30 cloned into the *Bam*HI/*Xba*I sites of pHT01; AmpR, CamR	This study
pAJW47	NEB10-beta pMA-T-*100AA-TEV*; fusion gene containing a small fraction of the cell wall binding domain of *B. subtilis lytC*, a 100 amino acid linker and the TEV detection module; AmpR	GeneArt/This study
pAJW52	NEB10-beta pMK-RQ-*lytC*$_{CWD}$*-100AA-TEV*; *Acc*I/*Spe*I fragment containing *100AA-TEV* from pAJW47 cloned into the same sites of pAJW17, replacing *5AA-TEV* with *100AA-TEV*; KanR	This study
pAJW57	NEB10-beta pHT01-*lytC*$_{CWD}$*-100AA-TEV*; *lytC-*$_{CWD}$*-100AA-TEV* from pAJW52 cloned into the *Bam*HI/*Xba*I sites of pHT01; AmpR, CamR	This study
pAJW66	NEB10-beta pSB3C5-*xylF*-B0032-*mCPX-CON*; Control sensor consisting of *xylF* promoter, B0032 RBS and *mCPX-CON*; CamR	This study
pAJW67	NEB10-beta pMA-T-*100AA-ELA*; fusion gene containing a small fraction of the cell wall binding domain of *B. subtilis lytC*, a 100 amino acid linker and the elastase detection module; AmpR	This study
pAJW68	NEB10-beta pMK-RQ-*lytC*$_{CWD}$*-100AA-ELA*; *Acc*I/*Spe*I fragment containing *100AA-ELA* from pAJW67 cloned into the same sites of pAJW17, replacing *5AA-TEV* with *100AA-ELA*; KanR	This study
pAJW71	NEB10-beta pHT01-*lytC*$_{CWD}$*-100AA-ELA*; *lytC-*$_{CWD}$*-100AA-ELA* from pAJW68 cloned into the *Bam*HI/*Xba*I sites of pHT01; AmpR, CamR	This study
pAJW105	NEB10-beta pMA-T-*100AA-CON*; fusion gene containing a small fraction of the cell wall binding domain of *B. subtilis lytC*, a 100 amino acid linker and the CON detection module; AmpR	This study
pAJW106	NEB10-beta pMK-RQ-*lytC*$_{CWD}$*-100AA-CON*; *Acc*I/*Spe*I fragment containing *100AA-CON* from pAJW105 cloned into the same sites of pAJW17, replacing *5AA-TEV* with *100AA-CON*; KanR	This study
pAJW110	NEB10-beta pHT01-*lytC*$_{CWD}$*-100AA-CON*; *lytC-*$_{CWD}$*-100AA-CON* from pAJW106 cloned into the *Bam*HI/*Xba*I sites of pHT01; AmpR, CamR	This study
***Bacillus subtilis* strains**		
WB800N	*nprE aprE epr bpr mpr*::*ble nprB*::*bsr Δvpr wprA*::*hyg cm*::*neo*; NeoR	MoBiTech GmbH; [3]
AJW5	WB800N pHT01; NeoR, CamR	This study
AJW6	WB800N pHT01- *lytC*$_{CWD}$*-5AA-TEV*; NeoR, CamR	This study
AJW10	WB800N pHT01-*lytC*$_{CWD}$*-50AA-TEV*; NeoR, CamR	This study
AJW15	WB800N pHT01-*lytC*$_{CWD}$*-100AA-TEV*; NeoR, CamR	This study
AJW22	WB800N pHT01-*lytC*$_{CWD}$*-100AA-ELA*; NeoR, CamR	This study
AJW23	WB800N pHT01-*lytC*$_{CWD}$*-100AA-CON*; NeoR, CamR	This study

Supplementary Table 6. Oligonucleotide primers used in this study

Number	Name	Sequence
**Primers for cloning**		
AJW64	5-inv-ELA	AGTTGGCCACTTGGCGGCC
AJW65	3-inv-ELA	TTGACCACTTTGACCGCGCAG
AJW66	5-inv-B0032	AGGAAAGTACTAGATGAAAAAAATTGCGTGCC
AJW67	3-inv-B0032	GTGTGACTCTAGTAATAAGTAACAATCACCGCG
AJW70	5-BamHI-lytC-fusion-pHT01	CCG**GGATCC**ATGCGTTCTTATATAAAAGTCC
AJW148	5-elast-100aa	CCACTAGGTGGCCAATCAGGACAACAT
AJW149	3-elast-100aa	CCAAGATTGTGATCCTTGCTGGCCTGA
AJW150	3-XbaI-LytC-100aa-pHT01	GGC**TCTAGA**TTATTAATGATGGTGATGATGATG
AJW180	5-control-QSG	CAGAGCGGTGGCGGCCAGAGCGGCCAGAGCG
AJW181	3-control-GSS	GCTGCTACCTTGACCACTTTGACCGCGCAGGC
AJW291	5-Con-100aa	CAATCAGGAGGTGGCCAATCAGGACAACAT
AJW292	3-Con-100aa	TGATGAGCCTTGTGATCCTTGCTGGCCTGA
**Primers for sequencing**		
AJW10	VF2	TGCCACCTGACGTCTAAGAA
AJW11	VR	ATTACCGCCTTTGAGTGAGC
AJW73	5-CWB-A	GGGGATAAGTATTAAACGAATTGC
AJW74	5-CWB-B	CCCTGACTCTATTGCAGGAGCTAC
AJW75	5-CWB-C	GCACCGTATATGTAAGCAATGG
AJW76	3-CWB-D	GGTGTTCCGCCTACAATAATTAC
AJW77	5-seq-pHT01	CAGCTATTGTAACATAATCGGTACG
AJW78	3-seq-pHT01	GCAGACAAAGATCTCCATGGACGC
AJW80	Sfor	GCCTCTTCGCTATTACGCCAG
AJW81	Stdrev	TTAGGCACCCCAGGCTTTAC

Relevant restriction sites in primer sequences are underlined and in bold

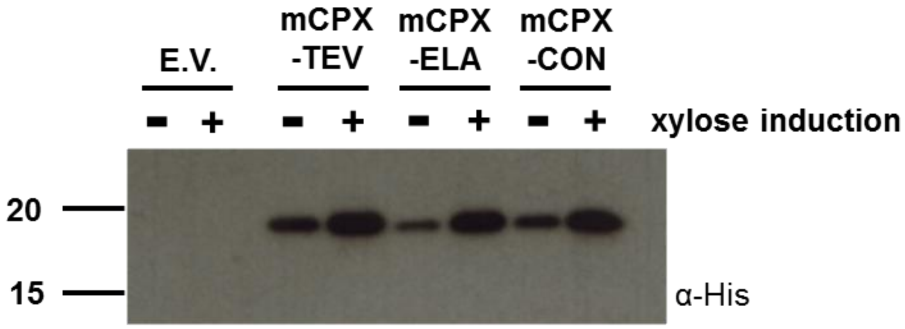

Supplementary Figure 1. *E. coli* based biosensors are expressed. Expression of the biosensors housed in *E. coli* cells. Cells were either induced (+) with 100 mM xylose or uninduced (-) for biosensor expression. Samples were separated on 12% PAA gels and the His-tagged biosensor proteins detected using an HRP-conjugated α-His antibody (1:4000 dilution). The predicted sizes of mCPX-TEV, mCPX-ELA and mCPX-CON after protein maturation and removal of the OmpX signal peptide is 20.8 kDa, 20.4 kDa and 20.5 kDa respectively. The positions of the protein molecular mass markers are indicated on the left in kDa.

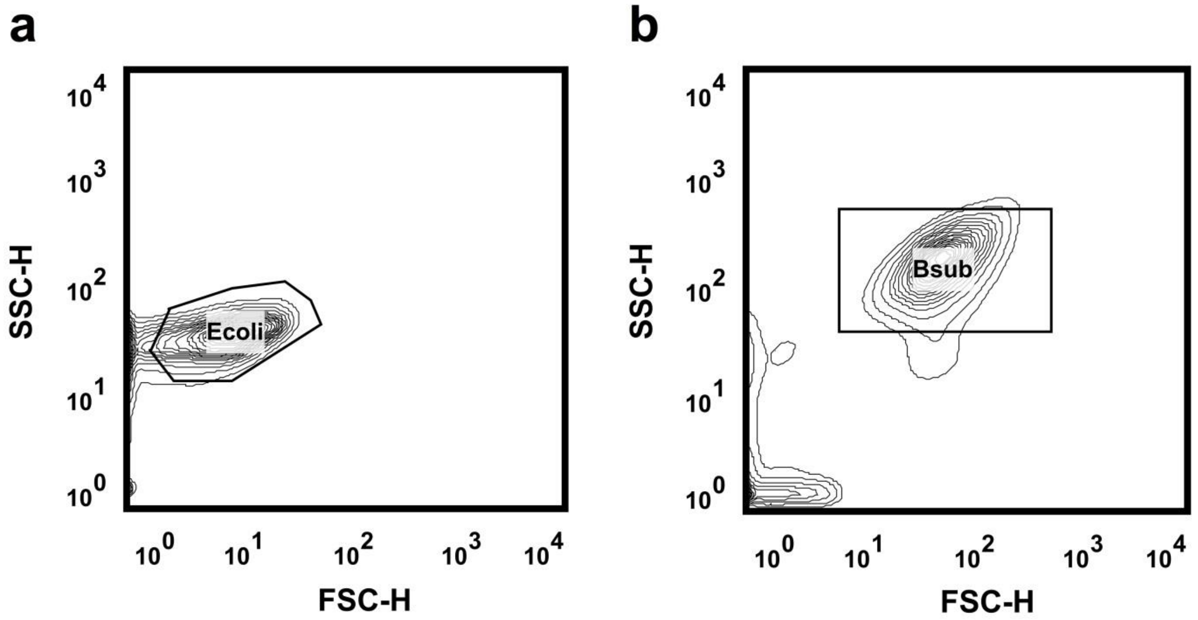

Supplementary Figure 2. Representative gating strategies used for flow cytometry analysis of (a) *E. coli* biosensors and (b) *B. subtilis* biosensors. Cell populations were gated based on forward scatter (FSC-H) and side scatter (SSC-H) analysis.

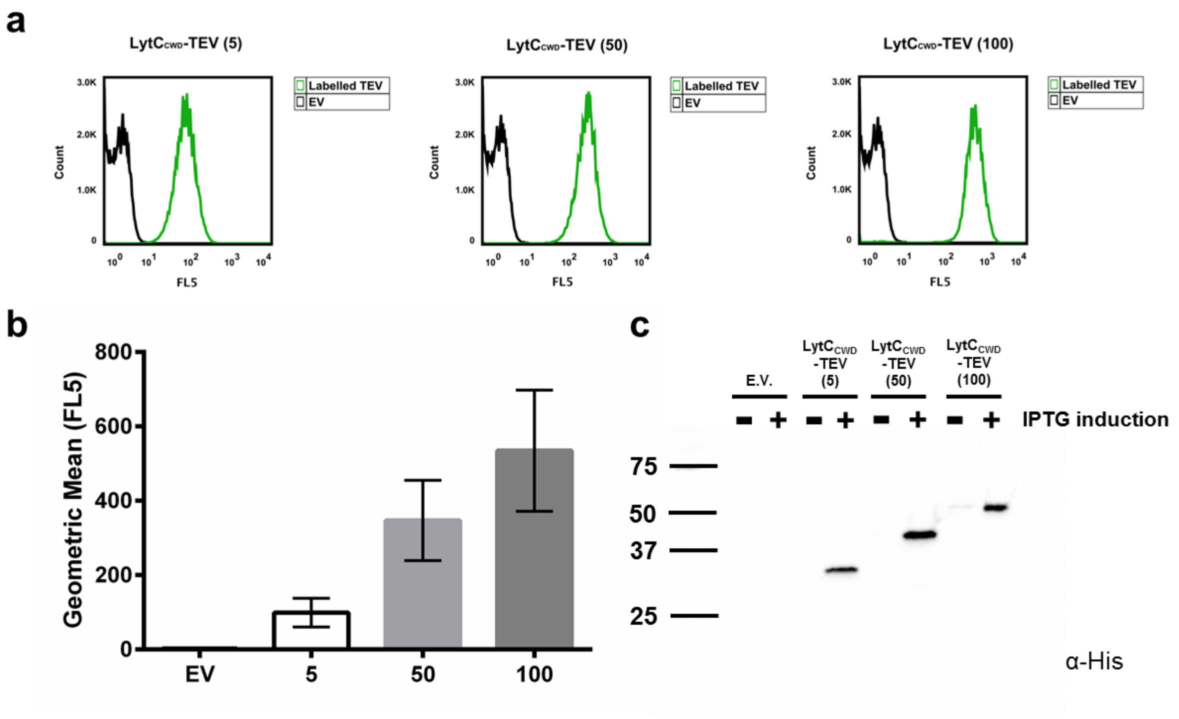

Supplementary Figure 3. Optimisation of linker length for maximum labelling of *B. subtilis* based TEV specific biosensors. (**a**) Flow cytometry analysis of cells expressing LytC$_{CWD}$-TEV biosensor with either a 5 (5), 50 (50) or 100 (100) amino acid linker before the detection module. A representative data set is shown. (**b**) Summary of flow cytometry data. EV is the empty vector control, whilst 5, 50 and 100 represent the LytC$_{CWD}$-TEV biosensor with either a 5, 50 or 100 amino acid linker respectively. Mean Geometric means were: LytC$_{CWD}$-TEV 5aa 99.2 ± 38.55, LytC$_{CWD}$-TEV 50aa 347 ± 108.06 and LytC$_{CWD}$-TEV 100aa 534.7 ± 163.13. (**c**) LytC$_{CWD}$-TEV biosensors with varying lengths are expressed at the correct size. Cells were either induced (+) with 1 mM IPTG or uninduced (-) for biosensor expression. Samples were separated on 12% PAA gels and the His-tagged biosensor proteins detected using an HRP-conjugated α-His antibody (1:4000 dilution). The predicted sizes of LytC$_{CWD}$-TEV (5aa), LytC$_{CWD}$-TEV (50aa) and LytC$_{CWD}$-TEV (100aa) after protein maturation and cleavage at the putative signal peptide are 33.2 kDa, 37.2 kDa and 41.7 kDa respectively. The positions of the protein molecular mass markers are indicated on the left in kDa.

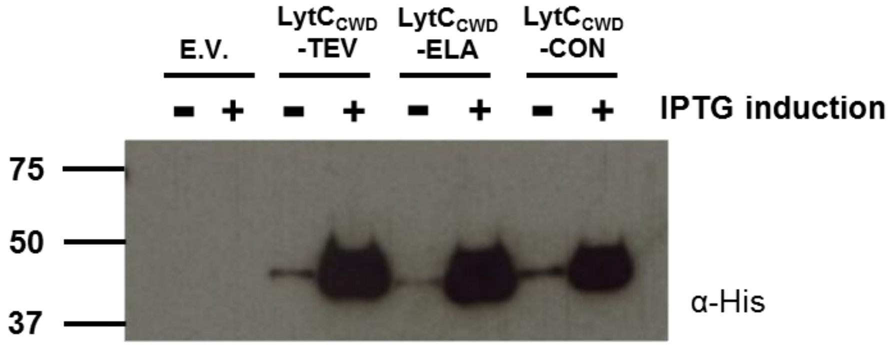

Supplementary Figure 4. *B. subtilis* based biosensors are expressed. Cells were either induced (+) with 1 mM IPTG or uninduced (-) for biosensor expression. Samples were separated on 12% PAA gels and the His-tagged biosensor proteins detected using an HRP-conjugated α-His antibody (1:4000 dilution). The predicted sizes of LytC$_{CWD}$-TEV, LytC$_{CWD}$-ELA and LytC$_{CWD}$-CON after protein maturation and cleavage at the putative signal peptide are 41.7 kDa, 41.3 kDa and 41.3 kDa respectively. The positions of the protein molecular mass markers are indicated on the left in kDa.

Supplementary References.

1. Kuroda, A. & Sekiguchi, J. Molecular cloning and sequencing of a major *Bacillus subtilis* autolysin gene. *Journal of Bacteriology* **173**, 7304-7312 (1991).
2. Nguyen, H. D., Phan, T. T. & Schumann, W. Expression vectors for the rapid purification of recombinant proteins in *Bacillus subtilis*. *Current Microbiology* **55**, 89-93, doi:10.1007/s00284-006-0419-5 (2007).
3. Nguyen, H. D., Phan, T. T. & Schumann, W. Analysis and application of *Bacillus subtilis* sortases to anchor recombinant proteins on the cell wall. *AMB Express* **1**, 22, doi:10.1186/2191-0855-1-22 (2011).
